# Supplementary material for: Early-life environment shapes claw bilateral asymmetry in the European lobster (Homarus gammarus)
Source: Biol Open. 2025 Mar 20;14(3):bio061901. doi: 10.1242/bio.061901 (PMC11957455; doi:10.1242/bio.061901)
Supplement: Supplementary information [file biolopen-14-061901-s1.pdf]

Table S1. Results of linear models (LMs) of the post hoc analyses for the Coefficient of asymmetry (Ca) in relation to treatment () across developmental stages (from 4th to 7th stage) for 244 individual European lobsters (*Homarus gammarus*). Reference levels are given in (). For statistically significant effects, p values are highlighted in bold.

Stage	Fixed factors	Estimate	SE	*t*	P
4th stage	Intercept	2.61000	0,55	4,73	<0.001
	Treatment (4SH)	-0.88265	0,77	-1,15	0,253
	Treatment (4SU)	0.35261	0,76	0,47	0,643
	**Treatment (5E)**	1.68733	0,76	2,22	**0,028**
	Treatment (5SH)	1.15969	0,82	1,41	0,161
	Treatment (5SU)	0.40080	0,79	0,51	0,614
	Treatment (6E)	0.28040	0,89	0,32	0,753
	Treatment (6SH)	-0.06507	0,81	-0,08	0,936
	Treatment (6SU)	-0.01762	0,82	-0,02	0,983
	Treatment (C)	0.98148	0,65	1,51	0,132
5th stage	Intercept	3,73	0,75	4,95	<0.001
	Treatment (4SH)	-1,03	1,07	-0,97	0,334
	Treatment (4SU)	-0,40	1,09	-0,37	0,712
	Treatment (5E)	-1,00	1,13	-0,88	0,380
	Treatment (5SH)	-2,32	1,19	-1,94	0,054
	Treatment (5SU)	1,68	1,11	1,52	0,130
	Treatment (6E)	0,39	1,11	0,36	0,722
	Treatment (6SH)	-1,31	1,16	-1,13	0,262
	Treatment (6SU)	0,19	1,16	0,17	0,868
	Treatment (C)	-0,47	0,90	-0,52	0,606
6th stage	Intercept	2,59	0,74	3,52	<0.001
	Treatment (4SH)	0,27	1,04	0,26	0,796
	**Treatment (4SU)**	3,40	0,97	3,52	**0,001**
	Treatment (5E)	0,68	1,12	0,61	0,541
	Treatment (5SH)	-1,02	1,19	-0,86	0,394
	Treatment (5SU)	1,79	1,25	1,44	0,152
	Treatment (6E)	0,42	1,09	0,39	0,697
	Treatment (6SH)	1,40	1,25	1,12	0,264
	Treatment (6SU)	1,83	1,12	1,64	0,105
	Treatment (C)	0,24	0,87	0,28	0,779
7th stage	Intercept	3,27	1,14	2,86	0,005
	Treatment (4SH)	0,26	1,50	0,18	0,861
	**Treatment (4SU)**	6,41	1,58	4,05	**<0.001**
	Treatment (5E)	1,49	1,66	0,90	0,371
	Treatment (5SH)	-0,30	1,78	-0,17	0,868
	**Treatment (5SU)**	4,23	1,72	2,47	**0,015**
	Treatment (6E)	1,14	1,72	0,66	0,509
	Treatment (6SH)	0,34	1,62	0,21	0,833
	**Treatment (6SU)**	4,69	1,58	2,97	**0,004**
	Treatment (C)	1,18	1,31	0,90	0,369

Biology Open • Supplementary information
